# Garlic Peel-Based Biochar Prepared under Weak Carbonation Conditions for Efficient Removal of Methylene Blue from Wastewater

**DOI:** 10.3390/molecules29194772

**Published:** 2024-10-09

**Authors:** Tao-Tao Shi, Bi Yang, Wei-Guo Hu, Guan-Jin Gao, Xin-Yu Jiang, Jin-Gang Yu

**Affiliations:** College of Chemistry and Chemical Engineering, Central South University, Changsha 410083, China222311019@csu.edu.cn (B.Y.); huweiguo@patlcell.com (W.-G.H.);

**Keywords:** organic dyes, adsorption, biochar, methylene blue, garlic peel

## Abstract

Background: Due to it containing cellulose, hemicellulose, and lignin with abundant specific functional groups which could interact with organic dyes, garlic peel (GP) might be used as an efficient biosorbent. The aim of this study is to evaluate the adsorption performances of GP-based bio-adsorbents and obtain optimum preparation conditions. Methods: GP-based bio-adsorbents were prepared by thermal pyrolysis under different temperatures (150–400 °C). The morphologies, chemical states, and surface functional groups of the adsorbents were analyzed by X-ray photoelectron spectroscopy (XPS), Fourier transform infrared spectroscopy (FT-IR), scanning electron microscopy (SEM), and thermogravimetric analysis (TGA). Batch experiments were conducted to investigate the adsorption of methylene blue (MB) under various conditions, including contact time, contact temperature, initial dye concentration, and initial pH value. The equilibrium adsorption data were fitted to different kinetic and isothermal models, and the adsorption thermodynamics were also calculated. Significant Findings: The physicochemical properties of the GP-based bio-adsorbents were primarily dominated by the pyrolysis temperature, because their morphologies and surface functional groups of GP-based bio-adsorbents significantly varied with the changes in pyrolysis temperature. The adsorption capacity of GP materials for MB decreased as the pyrolysis temperature increased. At an initial concentration of 50.00 mg L^−1^, GP150 possessed a higher adsorption capacity of 167.74 mg g^−1^ toward MB. The possible adsorbate–adsorbent interactions, including electrostatic attraction, hydrogen bonding, and π-π stacking, were recognized. After 10 consecutive adsorption–desorption cycles, GP150 maintained a high removal rate (88%) for MB, demonstrating its excellent adsorption performance, good reusability, and potential application in the treatment of MB-contaminated water.

## 1. Introduction

Due to the rapid development of modern industry and agriculture, an increasing amount of toxic organic substances are being discharged into the environmental water. Organic pollutants including dyes, pesticides, phenols, antibiotics, and so on are often resistant to natural degradation, therefore posing a significant threat to water ecosystems and human life [1]. It is crucial to develop effective methods for the management and treatment of these toxic, non-degradable pollutants. The removal of organic dyes from wastewater can be implemented through various methods, including biological oxidation [2], flocculation [3], chemical precipitation [4], and adsorption [5]. The effectiveness of contaminant removal varies depending on its species and the treatment strategy used. Among the aforementioned methods, the biological oxidation method has exhibited relatively lower removal efficiency; the flocculation and chemical precipitation methods have the potential to generate secondary pollutions. In comparison, the adsorption method has many advantages, including high efficiency, low cost, and being environmentally friendly and easy to operate. The adsorption method has been regarded as a simple, effective, and promising approach for the removal of organic dyes in wastewater [6].

In recent years, biosorbents derived from biomass have become a promising alternative for the removal of organic pollutants from wastewater due to their environmentally friendly properties, cost effectiveness, and high efficiency [7,8]. The most common biomaterials, such as plants, agricultural by-products, and municipal solid wastes, have been investigated. Biosorbents made from mango leaves [9], Korean cabbage [10], waste carton [11], and Retinervus luffae fructus [12] have demonstrated excellent adsorption performances toward organic dyes in aqueous solutions. Due to the increasing awareness of environmental protection and the continuous development of wastewater treatment technology, research on the use of agricultural by-products to prepare biochar for the removal of dye wastewater has been rapidly developed. Researchers are constantly exploring the preparation process, modification methods, and the joint application of biochar with other technologies in order to achieve more efficient and environmentally friendly wastewater treatment. Obviously, the adsorption efficiencies of a biosorbent for the contaminants are closely associated with its characteristics and the preparation techniques, and the rational selection and reasonable processing of a biomass with applicable components can achieve more efficient pollutant removal [13].

Garlic is a common condiment in the kitchen. Processed peeled garlic cloves can be used to prepare various commodities for sale, and the waste garlic peel (GP), accounting for 16–20% of the total amount of garlic, is produced in large quantities, which is almost 3 million tons annually [14]. Consequently, GP, an agricultural by-product, is widely accessible, cost-effective, and comprises a multitude of functional groups; thereby, it could reduce the expense of biochar and provide abundant active adsorption sites. GP is expected to serve as a suitable bio-adsorbent for the removal of dyes from water. The casual disposal of GP will cause the wastage of resources and unforeseen environmental threats. However, the species (cellulose, hemicellulose, and lignin) richness in GP makes it a potential bio-adsorbent due to the presence of various functional groups, which could interact with various contaminants [14]. For example, GP materials dried at 60 °C for 24 h exhibited a maximum adsorption capacity of 82.64 and 37.96 mg g^−1^ for MB [15] and Direct Red 12B [16], respectively. If GP materials were activated by Na_2_CO_3_, a maximum adsorption capacity of 52.6 mg g^−1^ for Congo red could be obtained [14]. Clearly, these GP-derived adsorbent materials have demonstrated relatively lower capacity and unsatisfactory adsorption effects. To preserve the functional groups naturally present in the biomass feedstocks that are beneficial to adsorption performances, including adsorption capacity as well as removal rate, low-temperature pyrolysis under vacuum or atmospheric conditions can be performed [17]. Compared to the thermal decomposition of organic components under atmospheric conditions, vacuum pyrolysis could offer a distinct advantage of shorter volatile residence time, which could prevent the pore plugging, inhibit the thermal decomposition of surface functional groups, and reduce the combustion reactivity of the biochar. Therefore, biochar with higher yield and quality would be produced [18].

Herein, we expect to investigate the effects of temperature on the physicochemical properties of GP-based bio-adsorbents by a vacuum pyrolysis procedure, achieve highly-efficient adsorption performance, and reveal the relationship between the “pyrolysis temperature–physicochemical properties of GP-adsorbent/adsorbate interactions”. A new attempt was made to obtain GP-based adsorbents that retain oxygen (O)- and nitrogen (N)-containing functional groups by vacuum low-temperature pyrolysis technology [19,20]. The GP materials were characterized by X-ray photoelectron spectroscopy (XPS), Fourier transform infrared spectroscopy (FT-IR), scanning electron microscopy (SEM), and thermogravimetric analysis (TGA), and the effects of adsorption condition parameters, such as contact time, contact temperature, initial dye concentration, and solution pH on the adsorption properties were investigated. The experimental data were fitted to various adsorption kinetic, isothermal, and thermodynamic models. The adsorption mechanism of GP materials for MB was proposed based on the structural characterizations, and the reusability of GP materials was evaluated. We hope to provide some experimental references for the rational carbonization of biomass sources to produce biochar with more excellent adsorption performances.

## 2. Results and Discussions

### 2.1. SEM

**Figure 1:**
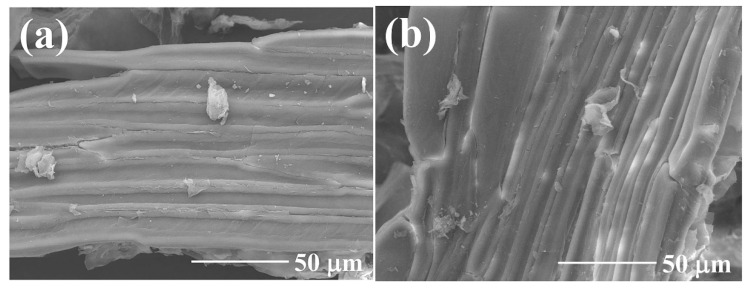
SEM images of GP150 material pre- (**a**) and post-adsorption (**b**) of MB.

SEM was used to investigate the surface morphology of GP150 pre- and post-adsorption of MB dye (Figure 1). The image of GP150 produced by low-temperature vacuum pyrolysis shows an irregular fibrous network structure, indicating that the basic cellulose structure remains unchanged (Figure 1a). The observation of the undamaged morphology of GP150 post-adsorption of MB dye confirms that GP150 could maintain its structure even after a long-time oscillating in aqueous solutions (Figure 1b).

**Figure 2:**
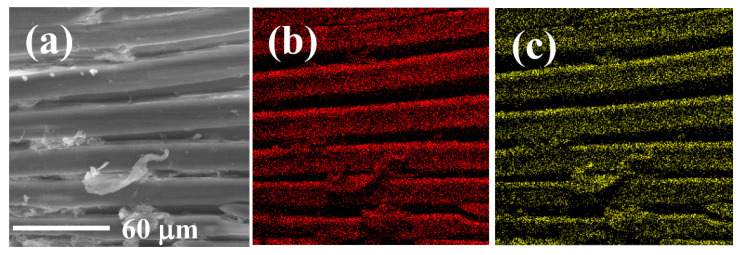

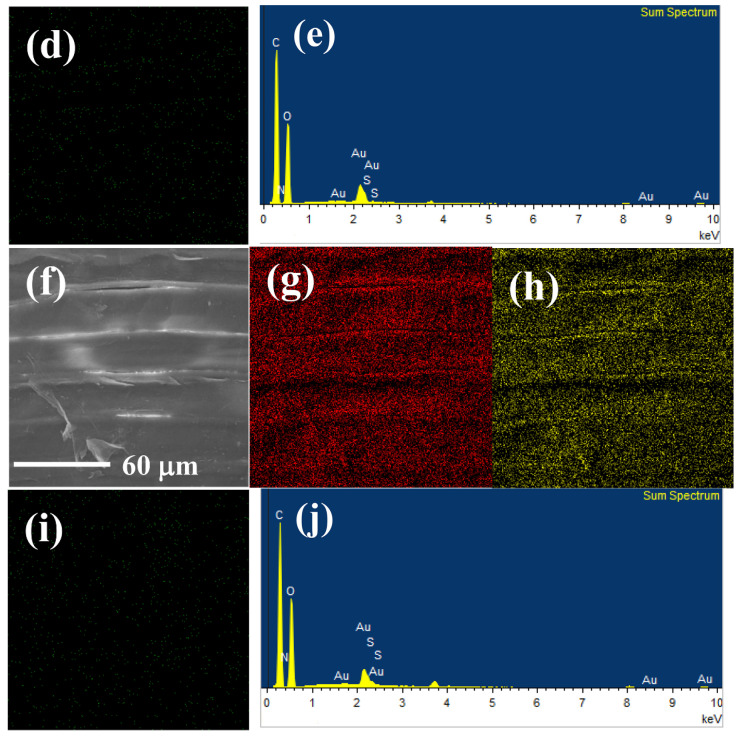
EDS and elemental mapping images of GP150: (**a**) SEM image of the scanned area; (**b**) C; (**c**) O; (**d**) N; (**e**) EDS of GP150; EDS and elemental mapping images of GP1500-MB: (**f**) SEM image of the scanned area; (**g**) C; (**h**) O; (**i**) N; (**j**) EDS.

EDS and elemental mapping analyses were implemented to observe the elemental distribution of GP150 composites pre- and post-adsorption of MB dye (Figure 2). Figure 2a shows the morphology of a tested region of the GP150 material pre-adsorption of MB. The homogeneous distribution of C (50.19 wt.%), N (0.55 wt.%), and O (42.90 wt.%) on GP150 could be observed (Figure 2b–e). After adsorption of MB (Figure 2f–j, there are some changes in the mass percentage of the elements on GP150-MB: C (49.89 wt.%), N (1.63 wt.%), O (42.84 wt.%), and S (0.44 wt.%). The effectiveness of the adsorption of MB dye by the GP150 material can be demonstrated by the increase in mass percentage of N and O elements, as well as the presence of the newly-emerging element S.

### 2.2. FT-IR Spectroscopy

**Figure 3:**
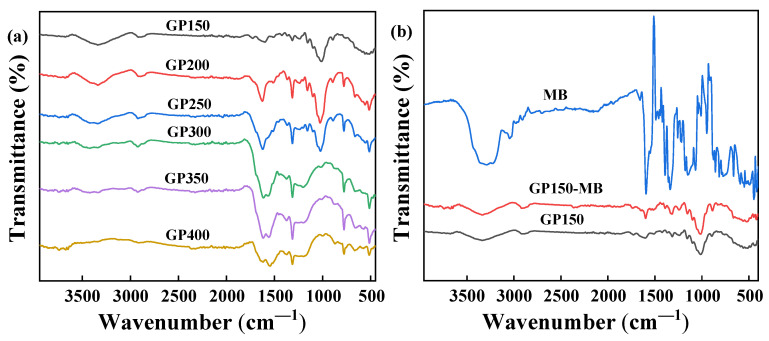
FT-IR spectra: (**a**) GP materials obtained by vacuum pyrolysis at different temperatures; (**b**) MB and GP150 pre- and post-adsorption of MB.

FT-IR spectroscopy was used to identify the changes in the functional groups on the surface of the GP materials. Figure 3a shows the FT-IR spectra of GP materials obtained at different pyrolysis temperatures, and the corresponding functional groups of GP materials are listed in Table 1 [21,22,23,24]. Obviously, the GP materials obtained through vacuum pyrolysis are rich in oxygen- and carbon-containing functional groups. In addition, significant differences could be found as the pyrolysis temperature increased, suggesting that some components (for example, O-H, C=O and C-O groups) of the GP materials are pyrolyzed and reformed during the heat-treatment process. The peak at 3339.11 cm^−1^ corresponds to the stretching vibration of the O-H group, which decreases significantly with increasing pyrolysis temperatures. In general, the presence of a large number of O-H groups promotes the adsorption reaction [25]. The peak at 1727.89 cm^−1^, corresponding to the stretching vibrations of C=O groups of aldehydes or ketones, noticeably decreases and is rapidly disappeared due to the pyrolysis at temperatures ≥250 °C. The peak at 1601.21 cm^−1^, corresponding to the stretching vibrations of -C=C- group, gradually increases, and the benzene skeletal vibrations at 1573.91 cm^−1^ appears at temperatures ≥300 °C, indicating that the carbonization of GP occurs at higher temperatures [26]. It is worth noting that the C-H bending vibrations of unsaturated C=C bonds at ~900 cm^−1^ decrease significantly with increasing pyrolysis temperatures, which is due to the pyrolysis and reforming of the C=C-H groups of the GP material forming more aromatic structures during the heat treatment.

The FT-IR spectra of MB dye and GP150 before and after the adsorption of MB dye are shown in Figure 3b. Obviously, the peak at 3339 cm^−1^ (N-H) of MB is greatly weakened, indicating that MB is adsorbed onto GP150 through the hydrogen bonding interactions. After the adsorption of MB onto GP150, the peak at 1665 cm^−1^ (-C=N) of MB disappears, while the peak at 1601.21 cm^−1^ (-C=C-) is enhanced, indicating that a π-π stacking interaction occurs between the GP150 material and MB dye [27].

### 2.3. XPS

**Figure 4:**
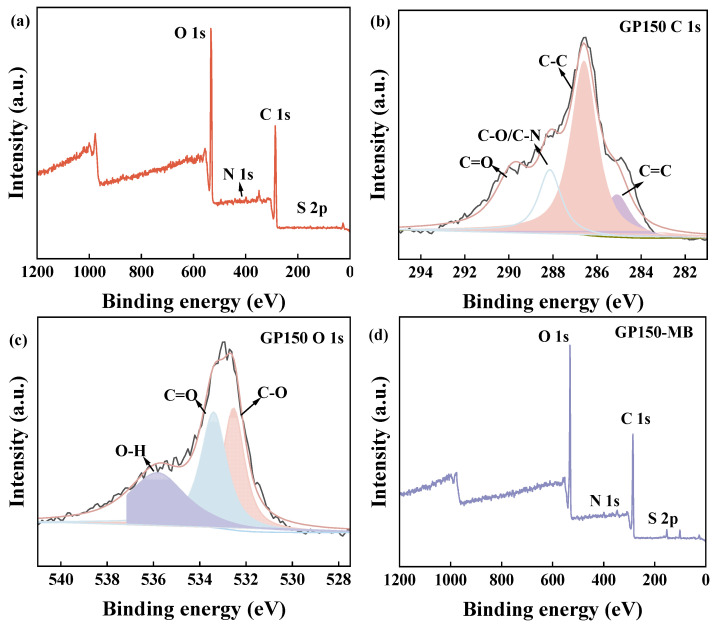

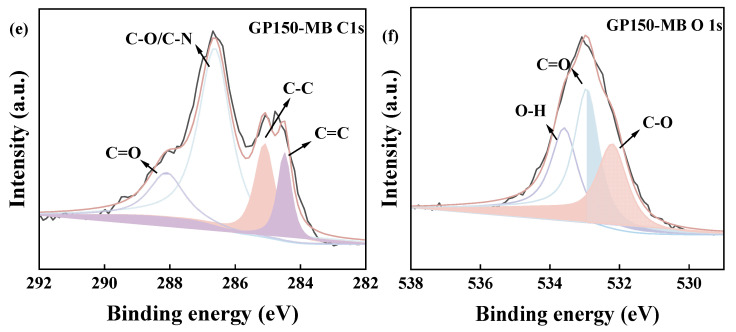
XPS spectra of GP150: (**a**) Survey spectra; XPS-peak-differentiation-imitating analyses of C 1s (**b**) and O 1s (**c**). XPS spectra of GP150-MB: (**d**) Survey spectra; XPS-peak-differentiation-imitating analyses of C 1s (**e**) and O 1s (**f**).

To analyze the surface properties of the GP materials, XPS was used to characterize GP150 and GP150-MB (Figure 4, Table 2). As evidenced by the increase in weight percentage of C, N, and S on the surface, the adsorption of MB dye by GP150 was confirmed (Table 2). The changes in bonding of GP150 pre- and post-adsorption of MB reveal the XPS-peak-differentiation-imitating analytic data of C 1s and O 1s, suggesting that the hydroxyl (-OH), carbonyl (C=O), and amino (C-N) groups present in the GP150 material play a key role in the adsorption process [28]. It is evident that the binding energies of the C- and O-based functional groups have changed, indicating that the adsorption of MB by GP150 material is an electron-acquiring process [29,30,31].

### 2.4. N_2_ Adsorption/Desorption Isotherms

**Figure 5:**
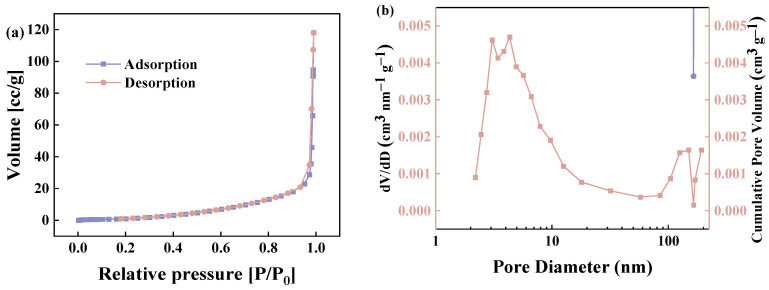
(**a**) N_2_ adsorption/desorption isotherms of GP150 material; (**b**) pore volume distribution of the GP150 material by the Barrett-Joyner-Halenda (BJH) method.

The specific surface area (SSA) and pore size distribution of the GP150 material were determined using N_2_ adsorption/desorption isotherms (Figure 5). The BET specific surface area, cumulative pore volume, and average pore size of the GP150 material were determined to be 5.46 m^2^ g^−1^, 0.18 cm^3^ g^−1^, and 148.65 nm, respectively. The GP150 material possesses low cumulative pore volume and large pores, which reconfirms its irregular fibrous network structure, and organic dyes would be mainly adsorbed by GP150 at the surface.

### 2.5. Adsorption Performances

**Figure 6:**
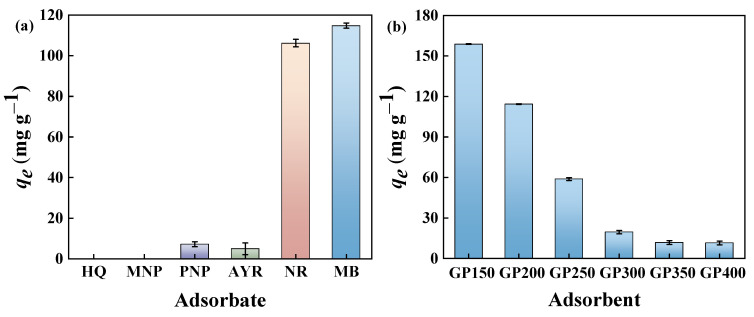
(**a**) Adsorption capacities of the GP200 material toward different dyes and phenols in aqueous solutions (*C*_0_ = 50 mg L^−1^, adsorbent dosage = 5.0 mg, contact time = 120 min, T = 25 °C, pH = at the natural pH of aqueous adsorbate solutions); (**b**) adsorption of MB by GP150, GP200, GP250, GP300, GP350, and GP400 (*C*_0_ = 50 mg L^−1^, adsorbent dosage = 5.0 mg, contact time = 120 min, T = 25 °C, pH = the natural pH of the aqueous adsorbate solution).

Batch adsorption experiments were carried out to investigate the adsorption performance of the GP200 material for various organic pollutants, including cationic dyes (MB), anionic dyes (AYR), neutral dyes (NR), and phenols (HQ, MNP, and PNP). The experimental results showed that the adsorption capacities of the GP200 material for HQ, MNP, PNP, AYR, NR, and MB were 0, 0, 7.16, 4.92, 106.19, and 114.41 mg g^−1^, respectively (Figure 6a). The results indicated that the GP200 material possessed a higher capacity for organic dyes, especially MB.

The effects of pyrolysis temperatures on the adsorption properties of GP materials (GP150, GP200, GP250, GP300, GP350, and GP400) for MB dye were investigated (Figure 6b). Clearly, the adsorption capacity of the GP materials for MB dye gradually decreases as the pyrolysis temperature increases, and the GP150 material exhibits the highest adsorption capacity. This is due to the reservation of abundant polar functional groups such as C=O, O-H, and C-N in the GP materials at low pyrolysis temperatures, which greatly facilitates the adsorption process. The results are in accordance with the FT-IR analyses, again suggesting the decrease in the amount and type of functional groups on the GP materials with increasing pyrolysis temperatures. In comparison with several previously reported adsorbents, the GP150 material exhibits a much higher adsorption capacity for MB at a lower initial concentration and adsorbent dosage (Table 3). To evaluate the adsorption performance of the GP150 material and reveal the adsorption mechanisms, MB was selected as a model pollutant to investigate the effect of experimental conditions on the adsorption procedure.

### 2.6. Effect of Adsorption Conditions

#### 2.6.1. Effect of Contact Time and Adsorption Kinetics

**Figure 7:**
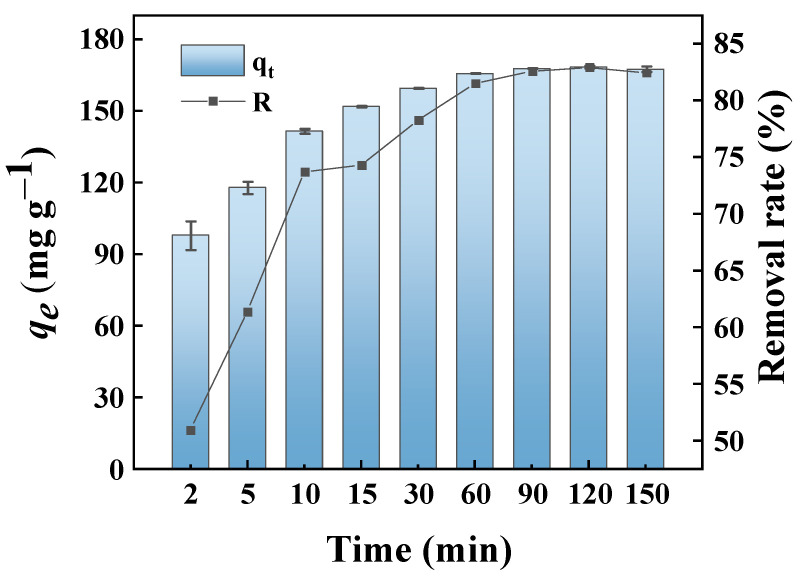
Effect of contact time on the removal of MB dye by the GP150 material (*C*_0_ = 50.0 mg L^−1^, T = 25 °C, pH = at the natural pH values of aqueous MB solutions).

Figure 7 shows the effect of contact time on the adsorption of MB by GP150 at an initial MB concentration of 50.0 mg L^−1^ at different time intervals (2–150 min). Clearly, the MB dye adsorbed on GP150 increases rapidly at the beginning of adsorption, then slows down, and finally reaches an adsorption equilibrium. During the initial stage of adsorption, the numerous active sites on GP150 could interact with MB dye, which has a high diffusion rate at high concentrations, causing a rapid adsorption process. Whereafter, the active adsorption sites of the GP150 material would be gradually occupied, accompanied by the gradual decrease of MB concentration in the solution; therefore, the adsorption rate gradually slows down and finally reaches equilibrium in 60 min [38]. The removal rate (%) and adsorption capacity of MB dye by the GP150 material at equilibrium are 83% and 168.41 mg g^−1^, respectively.

Adsorption kinetic models are very useful for estimating some essential parameters, and the experimental data could be evaluated with several known kinetic models [39]. To determine the kinetics of MB dye adsorption on the GP150 material, the data on the adsorption of MB dye by GP150 were fitted with the pseudo first-order (PFO) and the pseudo second-order (PSO) models (Figure S1, Table S3). The PSO model exhibits the highest coefficient of determination (*R*^2^ = 0.9751), and the calculated *q_e_* (q_e,cal_ = 169.18 mg g^−1^) is closer to the experimental data (q_e,exp_ = 168.41 mg g^−1^). Therefore, the pseudo second-order kinetic model could better predict the adsorption process of GP150 for MB than the pseudo first-order kinetic model. The plots of *q_t_* versus *t* for the adsorption of MB dye by GP150 do not pass through the origin, indicating that intra-particle diffusion is not the rate-controlling step in the adsorption process [40].

#### 2.6.2. Effect of Initial MB Concentration and Contact Temperature

**Figure 8:**
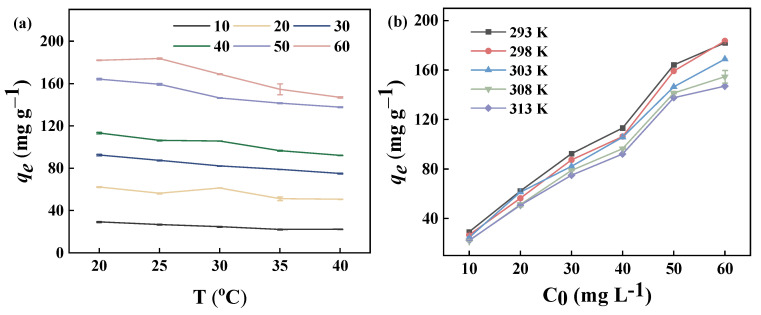
Adsorption properties of the GP150 material for MB: (**a**) effect of contact temperature (adsorbent dosage = 5.0 mg, *t* = 60 min, pH = the natural pH values of the dye stuffs); (**b**) effect of initial MB concentration (adsorbent dosage = 5.0 mg, *t* = 60 min, pH = the natural pH values of the dye stuffs).

The contact temperature and the initial concentration of the dye are the key factors affecting the adsorption process. In general, the diffusion rate of the adsorbent is also influenced by the temperature. The concentration of the adsorbent plays a crucial role in determining the diffusion rate, which in turn affects the efficiency of adsorption and the removal rate. Figure 8a shows the effect of contact temperature on the adsorption properties of the GP150 material. At the same initial dye concentration, the adsorption capacity of the GP150 material for MB dye slightly decreases with increasing contact temperature. As temperature increases, the intermolecular interaction forces between the adsorbent and the adsorbate are weakened, causing a decrease in the adsorption capacity of the GP150 material for MB dye [41].

The effect of initial MB dye concentration on the adsorption capacity of GP150 is shown (Figure 8b). The adsorption capacity of the GP150 material for MB dye is significantly affected by the initial dye concentration. At low initial MB concentrations, the active adsorption sites of the GP150 material are not fully occupied. However, the increase in the MB concentration resulting could provide a significant incentive to overcome the mass-transfer resistance between the aqueous solution and the solid adsorbent [42]. Therefore, a higher initial dye concentration would accelerate the adsorption of MB dye by the GP150 material, and a higher adsorption capacity would be observed with an increasing initial dye concentration.

#### 2.6.3. Adsorption Isotherms

Adsorption isotherms can be used to describe and explain the relationship between an adsorbent and an adsorbate [43]. The experimental adsorption equilibrium data were analyzed using the Langmuir and the Freundlich isothermal models, and the applicability of the models was determined and compared by evaluating the statistical indicators. The Langmuir isotherm model is used to determine adsorption of adsorbates on monolayer and specific homogeneous surface adsorbent surfaces, while the Freundlich isotherm model assumes that a multilayer adsorption occurs on the heterogeneous surfaces [44]. The adsorption isothermal constants could be calculated from Equations (S4) and (S5) by fitting the adsorption data, and the adjustable parameters in the Langmuir and Freundlich isotherms are estimated by the linear regression. The higher coefficient of determination (*R*^2^) suggests that the Freundlich model can better describe the adsorption of MB dye on the GP150 material (Figure S2, Table S4). The Freundlich model describes non-ideal and reversible adsorption processes that may form multiple layers on heterogeneous surfaces with uneven heat distribution. The adsorption of MB by the GP150 material is a complex process; the weak interactions between the adsorbent may also interfere with the adsorption process [45]. In the Freundlich model, K_F_ is related to the adsorption capacity, and *n* is related to the adsorption strength of the adsorbent [46,47]. The *n* of the GP150 material for the adsorption of MB dye is >1, indicating that it has excellent adsorption characteristics for MB dye.

#### 2.6.4. Adsorption Thermodynamics

In order to determine the thermodynamic parameters of MB adsorption on GP150, the curves between $InK_{eq}^{{}^{\circ}}$ and 1/T were plotted (Figure S3), and the calculations of Δ*H*°, Δ*S*°, and Δ*G*° were carried out by Equations (S6)–(S8). Table S5 illustrates the thermodynamic parameters obtained by the linear regression. The negative Δ*H*° indicates that the adsorption of MB dye on the GP150 material is exothermic. The Δ*H*° value calculated from the thermodynamic parameters could reveal whether physical or chemical adsorption occurs between the adsorbate molecules and the adsorbent surface [48]. The Δ*H*° value of a physical adsorption is generally in the range 2.1–40 kJ mol^−1^, while the Δ*H*° value of a chemical adsorption is generally in the range of 40–200 kJ mol^−1^ [49]. The Δ*H*° value for the adsorption of MB dye on the GP150 material is <40 kJ mol^−1^, suggesting physical adsorption occurs. The negative value of Δ*S*° (−80.563 J mol^−1^ K^−1^) indicates that the adsorption of MB on GP150 is an entropy decreasing process. During the adsorption process, MB occupies the active site in the GP150 material, resulting in ordered assembly behavior. This leads to a decrease in both the intramolecular and intermolecular degrees of freedom of MB molecules at the solid–liquid interface, resulting in a decrease in entropy value.

### 2.7. Effect of pH

**Figure 9:**
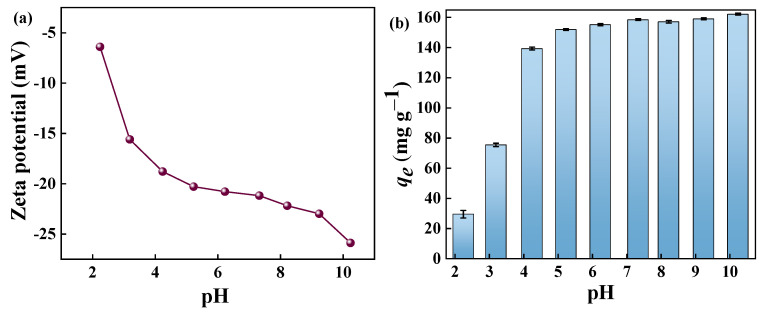
(**a**) Zeta potential of GP150 in aqueous solution at different pH values; (**b**) effect of initial solution pH value (*C*_0_ = 50.0 mg L^−1^, *t* = 60 min, T = 25 °C).

The solution pH value is an important parameter that affects the charge distribution on the adsorbent surface and the interactions between the adsorbent and the adsorbate [50]. Therefore, the charge properties of the adsorbent in the pH range 2–10 were investigated using zeta potential analyses. The solution pH values were adjusted with 0.1 mol L^−1^ NaOH and 0.1 mol L^−1^ HCl. As shown in Figure 9a, GP150 is significantly negatively charged, and an increase in the solution pH in the range of 2–10 produces more negative potentials. The effect of solution pH on the adsorption MB of GP150 was investigated (Figure 9b). Under acidic conditions, the adsorption capacity of the GP150 material for MB was relatively low, which gradually increased with the increase of the solution pH. This can be explained by the protonation and deprotonation of active sites in response to the solution pH. Under acidic conditions, the surface of GP150 undergoes protonation. Under alkaline conditions, the surface of GP150 undergoes deprotonation, which facilitates the interaction of GP150 with MB, a cationic dye. The surface charge density increases with the increasing solution pH, causing an enhanced electrostatic effect between the adsorbent and the adsorbate.

### 2.8. Adsorption of MB in Real Samples

**Figure 10:**
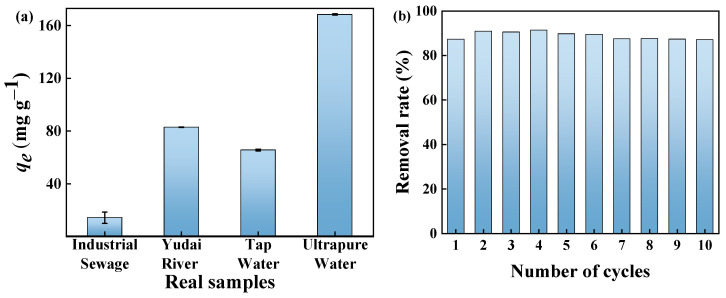
(**a**) The adsorption of MB from actual water samples by GP150 (*C*_0_ = 50.0 mg L^−1^, *t* = 60 min, T = 25 °C, pH = the natural pH values of the dye stuffs); (**b**) reusability of GP150 for removal of MB in successive 10 adsorption–desorption cycles (C_0_ = 20 mg L^−1^, adsorbent dosage = 50.0 mg, t = 60 min, T = 25 °C, pH = the natural pH values of the dye stuffs).

In order to evaluate the adsorption performance of the GP150 material for MB dye in real water samples, 50 mg L^−1^ of MB dye solution was prepared using ultrapure water, industrial wastewater, tap water, and pond water, respectively. Clearly, the adsorption capacity of GP150 for MB dye decreases in industrial wastewater (14.33 mg g^−1^), tap water (65.63 mg g^−1^), and Yudai River water (82.92 mg g^−1^) in comparison with ultrapure water (Figure 10a). Due to the competition of effective adsorption sites on the surface of the material by the co-existing contaminants (for example, various metal ions such as Zn^2+^, Pb^2+^, Fe^3+^, and Ca^2+^ present in industrial wastewater), varying degrees of decrease in the adsorption capacity of the GP150 material occur.

### 2.9. Reusability of GP150

Recyclability is an important feature of a newly developed adsorbent, which reflects the stability and practicality of the adsorbent to a certain extent, and higher reusability also demonstrates its more cost-effective property. A 50.0 mg quantity of the GP150 material was added to 20.0 mL of MB solution (50 mg L^−1^) and oscillated at 25 °C for 60 min, and then MB dye was desorbed from the GP150 material with 40.0 mL of HCl (0.1 mol L^−1^) under oscillation conditions. After a facile filtration, the GP150 material was recovered and used directly in the next adsorption–desorption cycle. The results indicate that even after 10 adsorption–desorption cycles (Figure 10b), the GP150 material maintains a high removal rate for MB dye (88%), demonstrating its excellent stability and good recoverability.

### 2.10. Adsorption Mechanisms

**Figure 11:**
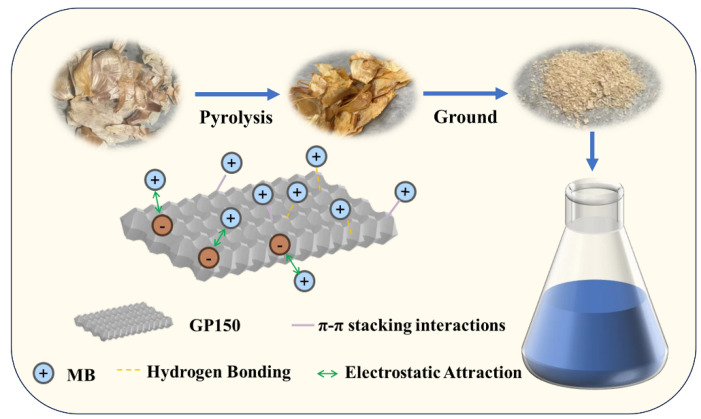
A proposed mechanism of MB adsorption by the GP150 material.

The adsorption mechanism of the GP150 material for MB dye is proposed. MB is a cationic dye that contains aromatic and heterocyclic rings, and its positively charged ammonium ion significantly affects the intermolecular interaction forces between MB and the GP150 material. The possible interactions between the GP150 material and the MB dye may include hydrogen bonding and π-π stacking, which were confirmed by FT-IR and XPS results, as previously discussed. Furthermore, the electrostatic interaction was revealed by investigating the effect of pH on the adsorption process. Therefore, a possible adsorption mechanism is proposed and presented in Figure 11.

## 3. Materials and Methods

### 3.1. Chemicals and Reagents

Garlic was purchased from a local market in Changsha, China, and GP was collected and naturally dried under room temperature. Physicochemical characteristics of organic dyes (MB, Alizarin Yellow R (AYR), and Neutral Red (NR)) and phenols (hydroquinone (HQ), p−nitrophenol (PNP), and 3−nitrophenol (MNP)) are shown in Tables S1 and S2, which were bought from Sinopharm Chemical Reagent Co., Ltd. (Shanghai, China). In addition, hydrochloric acid (HCl, 37.0 wt.%) was provided by Chengdu Colon Chemicals Co., Ltd. (Chengdu, China). Sodium hydroxide (NaOH, AR) was purchased from Hunan Huihong Reagent Co, Ltd. (Changsha, China). All chemicals in this study are analytical grade and used without further purification.

### 3.2. Preparation of GP-Based Adsorbents

Garlic peel (GP) was washed with ultrapure water to remove impurities and then dried in an oven at 60 °C for 48 h. After being cut into pieces, 1.0 g of the sample was placed in a porcelain combustion boat. The boat was wrapped with tin foil and subjected to the vacuum-heat treatment in a tube furnace, then heated to various temperatures (150 °C, 200 °C, 250 °C, 300 °C, 350 °C, and 400 °C) for 120 min with a heating rate of 5 °C min^−1^, and the obtained GP materials were defined as GP150, GP200, GP250, GP300, GP350, and GP400, respectively. The materials were further ground into powders, sieved, and washed twice with ultrapure water and three times with anhydrous ethanol. The materials were then dried in an oven at 70 °C for 180 min, then transferred to a desiccator for future use.

### 3.3. Characterization of the Materials

The produced GP150 materials were characterized using X-ray photoelectron spectroscopy (XPS), Fourier transform infrared spectroscopy (FT-IR), scanning electron microscopy (SEM), thermogravimetric analysis (TGA), and surface area analysis based on the Brunauer–Emmett–Teller (BET) method. The surface morphology of GP materials pre- and post-adsorption of dye molecules was observed by SEM (JEOL, JSM-6360LV; Tokyo, Japan). XPS was utilized to analyze the elemental composition and bonding patterns of GP materials. FT-IR spectroscopy can be used to detect the changes in the functional groups of GP materials pre- and post-adsorption of MB dye. The specific surface areas and pore sizes of the GP materials were determined through N_2_ adsorption/desorption isotherms using BET and Barrett–Joyner–Halenda (BJH) methods on a Kubo X1000 surface area detecting instrument analyzer (Beijing Builder Electronic Technology Co., Ltd.; Beijing, China), respectively.

### 3.4. Adsorption Experiments

Typically, 5.0 mg of GP materials and 20.0 mL of dyes (MB, AYR, and NR) and phenols (HQ, MNP, and PNP) were added to a 100 mL conical flask. The flask was then subjected to constant oscillation in a thermostatic oscillator at specific temperatures (15 °C, 20 °C, 25 °C, 35 °C, and 45 °C) for a certain period of time (0–180 min). A 752 UV-Vis spectrophotometer (Shanghai Jinghua Technology Instruments Co., Ltd.; Shanghai, China) was used to determine the concentrations of different organic contaminants (MB: λ_max_ = 664 nm; AYR: λ_max_ = 370 nm; NR: λ_max_ = 542 nm; PNP: λ_max_ = 318 nm; HQ: λ_max_ = 289 nm; MNP: λ_max_ = 327 nm) pre- and post-adsorption (Table S6).

In order to evaluate the adsorption performance of the prepared GP150 material for MB dye, batch adsorption experiments were carried out. The effects of various conditional parameters, including contact time, solution pH, initial dye concentration, and contact temperature on dye removal, were investigated. In order to evaluate the adsorption performance of GP150 in real water samples, ultrapure water, industrial wastewater, tap water and pond water were selected for the experiments. In addition, 10 adsorption–desorption cycle experiments were performed on the GP150 material in order to evaluate its reusability. All the adsorption experiments were parallelly carried out three times to improve the accuracy of the experimental results.

The equilibrium adsorption capacity (*q_e_*, mg g^−1^) and removal rate (*R*, %) of GP material composites for various adsorbates can be calculated by the following equations:

(1) $$q_{e}=\frac{(C_{0}-C_{e})\cdot V}{m}$$

(2) $$R=\frac{C_{0}-C_{e}}{C_{0}}\times 100\%$$

where *C*_0_ (mg L^−1^) is the initial adsorbate concentration, *q_e_* (mg g^−1^) is the equilibrium adsorption capacity of the adsorbent, *C_e_* (mg L^−1^) is the equilibrium adsorbate concentration, and *V* (L) and *m* (g) are the volume of adsorbate and the mass of GP materials, respectively.

## 4. Conclusions

In this study, a facile and environmentally friendly method was used to prepare the GP150 material, which can be used as an efficient and low-cost adsorbent for MB dye removal. An adsorption mechanism was proposed by analyzing the GP150 material pre- and post-adsorption of MB. The adsorption of MB dye onto the GP150 material was found to be mainly facilitated by the electrostatic, hydrogen bonding, and π-π stacking interactions. The adsorption results showed that the newly developed adsorbent is an efficient adsorbent with a high adsorption capacity. In addition, the thermodynamic analyses suggested that the adsorption is a spontaneous and exothermic process. After 10 adsorption–desorption cycles, the GP150 material shows high removal rate (88%) for MB dye, indicating it possesses high stability and good reusability. The adsorption performance of GP150 for MB dye in real samples varies to a certain extent but is still acceptable. Therefore, the GP150 material might have broad application prospects for the practical treatment of organic dye-containing wastewater in the future.

## Figures and Tables

**Table 1:** Major functional groups of GP materials with different pyrolysis temperatures.

Kind of Vibrations	Wavenumber (cm^−1^)					
	GP150	GP200	GP250	GP300	GP350	GP400
ν (O-H)	3339.11	3339.93	3341.32	3420.98	3421.34	3342.41
ν(C-H)	2916.85	2896.30	2920.12	2923.02	2923.63	2910.37
ν (C=O)	1727.89	−	−	−	−	−
ν (C=C)	1601.21	1627.20	1624.28	1616.50	1619.12	1619.38
Skeletal vibration	−	−	−	1573.91	1560.44	1549.27
ν (C-O)	1011.58	1026.69	1022.45	1020.76	1010.95	1004.06

Note: “−“: Not observed.

**Table 2:** Quantitative characterization of the surface elemental compositions of GP150 and GP150-MB by XPS.

Sample		GP150		GP150-MB	
			Binding Energy (eV)		Binding Energy (eV)
Element (wt.%)	C	56.2	−	58.4	−
	N	0.8	−	1.1	−
	O	42.9	−	40.3	−
	S	0.2	−	0.3	−
C	C=O	20.62	298.81	14.86	288.10
	C-O/C-N	15.71	288.18	55.55	286.63
	C-C	51.06	286.60	17.72	285.11
	C=C	12.61	285.09	11.88	284.47
O	C-O	31.94	535.85	27.95	533.61
	O=C	36.06	533.37	40.95	532.94
	H-O	32.00	532.52	31.46	532.22

Note: “−“: Not observed.

**Table 3:** A comparison of adsorption performance of GP150 for MB with other previously reported adsorbents.

Adsorbent	Activated Method/Activator	Adsorbent Dosage	*C*_0_ (mg L^−1^)	*q*_max_ (mg g^−1^)	Ref.
Heavy bio-oil	Furnace (800 °C, N_2_)	0.1 g/0.05 L	1000	411	[32]
Eggshell membranes	KOH and HNO_3_/Furnace (200 °C, N_2_)	0.025 g/0.02 L	100	110.38	[33]
Rice straw	Toxicity characteristic leaching procedure/Furnace (500 °C, N_2_)	1.0 g/1.0 L	1000	51.34	[34]
Cardboard waste	Pyrolysis technique (INRS—ETE (Quebec (Qc), Canada))	0.5 g/0.05 L	50	16.30	[6]
Fructus Aurantii Immaturus residues	NaOH/Furnace (750 °C, N_2_)	0.02 g/0.025 L	400	298.90	[35]
Eucheuma cottonii seaweed	Electric oven/H_2_SO_4_ (70 °C 48 h)	0.3 g/1.0 L	200	133.33	[36]
Banana pseudostem	Microwave (0.8 kW 20 min)	1.0 g/0.03 L	1000	165.5	[37]
GP150	Vacuum tube furnace (150 °C)	0.005 g/0.02 L	50	167.74	This work

## Data Availability

Data are contained within the article and Supplementary Materials.

## Supplementary Materials

The following supporting information can be downloaded at: https://www.mdpi.com/article/10.3390/molecules29194772/s1, Table S1: Properties and structures of MB and AYR and NR dyes. Table S2: Properties and structures of phenols (HQ, MNP, and PNP). Figure S1: (a) Fitted adsorption kinetic curves by nonlinear pseudo-first-order models; (b) Fitted adsorption kinetic curves by nonlinear pseudo-second-order models. Table S3: Adsorption kinetic parameters for the adsorption of MB onto GP150. Figure S2: (a) The experimental data of GP150 toward MB and the fitting curves of Langmuir isotherm model; (b) The experimental data of GP150 toward MB and the fitting curves of Freundlich isotherm model. Table S4: Adsorption isothermal parameters for the adsorption of MB onto GP150. Figure S3: Experimental data and the fitted curve of $InK_{eq}^{{}^{\circ}}$ versus 1/T calculated from van’t Hoff plots of adsorption of MB onto GP150. Table S5: Thermodynamic parameters of the adsorption of MB onto GP150. Table S6: Calibration curves of MB at different pH values.
