# Supplementary material for: Garlic Peel-Based Biochar Prepared under Weak Carbonation Conditions for Efficient Removal of Methylene Blue from Wastewater
Source: Molecules. 2024 Oct 9;29(19):4772. doi: 10.3390/molecules29194772 (PMC11478232; doi:10.3390/molecules29194772)
Supplement: Supplementary file 1 [file molecules-29-04772-s001.zip › molecules-3215277-supplementary.pdf]

Garlic peel-based biochar prepared under weak carbonation conditions for efficient removal of methylene blue from wastewater

Tao-Tao Shi, Bi Yang, Wei-Guo Hu, Guan-Jin Gao, Xin-Yu Jiang, Jin-Gang Yu*

College of Chemistry and Chemical Engineering, Central South University, Changsha, Hunan 410083, China

1. Physico-chemical characteristics of dyes and phenols

Table S1 Properties and structures of MB and AYR and NR dyes

Dyes	Methylene blue (MB)	Alizarin Yellow R (AYR)	Neutral Red (NR)
**Molecular formula**	$C_{16}H_{18}ClN_3S$ 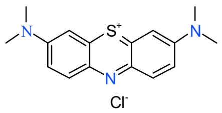	$C_{13}H_8N_3O_5Na$ 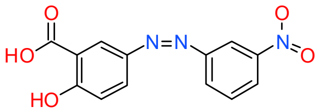	$C_{15}H_{17}ClN_4$ 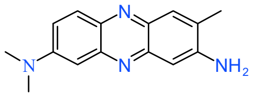
**Molecular weight (g mol^{-1})**	319.85	287.23	288.77
**Classification**	cationic dye	anionic dye/azo dyes	neutral dye
**CAS**	61-73-4	2243-76-7	553-24-2
**Melting point (K)**	463	523	563
**λ_{max} (nm)**	664	370	542

Table S2 Properties and structures of phenols (HQ, MNP, and PNP).

Phenols	Hydroquinone (HQ)	3-nitrophenol (MNP)	p-nitrophenol (PNP)
**Molecular formula**	$C_6H_6O_2$ 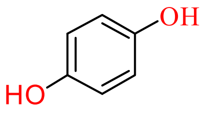	$C_6H_5NO_3$ 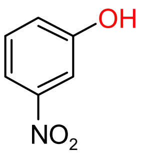	$C_6H_5NO_3$ 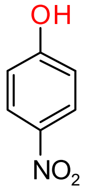
**Molecular weight (g mol^{-1})**	110.11	139.11	139.11
**CAS**	204-617-8	209-073-5	100-02-7
**Melting point (K)**	447	370	386
**λ_{max} (nm)**	289	327	318

2. Adsorption kinetics

To whom correspondence should be addressed.

* JG Yu, E-mail: yujg@csu.edu.cn; Tel/Fax: +86-731-88879616.

Two couples of kinetic models were used to fit the experimental data of contact time on MB adsorbed onto GP150. The first models, nonlinear pseudo-first-order models, could be expressed as follows:

$$q_t = \frac{(C_0 - C_t) \cdot V}{m} \quad \textbf{(S1)}$$

$$q_t = q_e(1\text{-}e^{-k_1 t}) \quad \textbf{(S2)}$$

The second models, nonlinear pseudo-second-order models, could be expressed as follows:

$$q_t = \frac{k_2 q_e^{\ 2} t}{1 + k_2 q_e t} \quad \textbf{(S3)}$$

where t (min) is the contact time; q_e (mg g^{-1}) and q_t (mg g^{-1}) are the amounts of MB adsorbed at equilibrium and at time t, respectively; k_1 (min^{-1}) and k_2 (g·$mg^{-1}min^{-1}$) are the specific adsorption rate constants of the pseudo first-order model and the pseudo second order models, respectively.

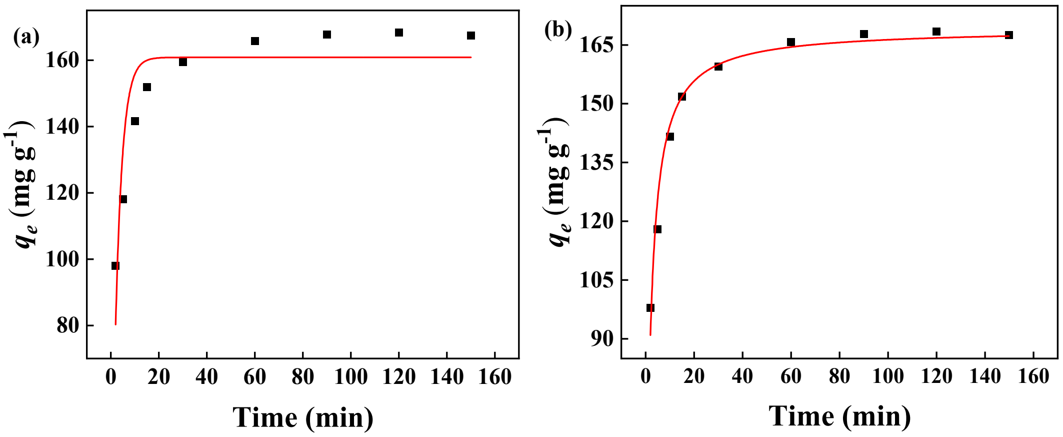

Figure. S1 (a) Fitted adsorption kinetic curves by nonlinear pseudo-first-order models; (b) Fitted adsorption kinetic curves by nonlinear pseudo-second-order models.

Table S3 Adsorption kinetic parameters for the adsorption of MB onto GP150.

Fitting parameters	Non-linear pseudo-first-order model	Non-linear pseudo-second-order model
R^2	0.8116	0.9751
k_1 (min^{-1})	0.3449	-
k_2 (g $mg^{-1}min^{-1}$)	-	0.0034
q_e (mg g^{-1})	160.85	169.18

3. Adsorption isotherms and thermodynamics

Two isothermal models were used to fit the experimental data of MB adsorbed on GP150.

Langmuir model:

$$q_e = \frac{q_m K_L C_e}{1 + K_L C_e} \quad \textbf{(S4)}$$

where C_e (mg L^{-1}) is the equilibrium concentration; q_e (mg g^{-1}) is the amount of adsorbed species per specified amount of adsorbent; K_L (L mg^{-1}) is the Langmuir equilibrium constant and q_m (mg g^{-1}) is the amount of adsorbate required to form an adsorbed monolayer.

Freundlich model:

$$q_e = K_F C_e^{\frac{1}{n}} \quad \textbf{(S5)}$$

where C_e (mg L^{-1}) is the equilibrium concentration of adsorbent; q_e (mg g^{-1}) is the amount of adsorbent per unit mass; K_F (mg $L^{1/n}$ g^{-1} $mg^{-1/n}$) and n are two Freundlich constants: n represents the relative advantage of adsorption process; K_F is the affinity constant which is related to the adsorption capacity of the adsorbent and can also be defined as adsorption or distribution coefficient, indicating the amount of dye adsorbed on GP150 at equilibrium.

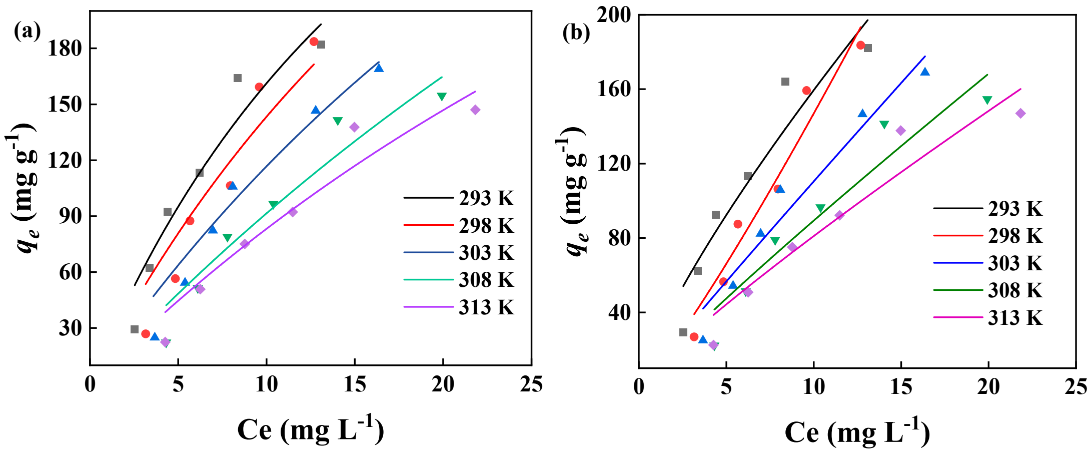

Figure. S2 (a) The experimental data of GP150 toward MB and the fitting curves of Langmuir isotherm model; (b) The experimental data of GP150 toward MB and the fitting curves of Freundlich isotherm model.

Table S4 Adsorption isothermal parameters for the adsorption of MB onto GP150

**Model**	**Fitting parameters**	**293 K**	**298 K**	**303 K**	**308 K**	**313 K**
**Langmuir**	R^2	0.9270	0.8910	0.9408	0.9314	0.9291
	K_L (L mg^{-1})	0.434	0.0289	0.0198	0.0152	0.0121
	q_m (mg g^{-1})	523.40	638.85	704.70	627.95	848.57
**Freundlich**	R^2	0.9056	0.9534	0.9515	0.9203	0.9192
	K_F (mg L$^{1/n}$ g^{-1} mg$^{-1/n}$)	25.8739	10.3001	11.9059	10.8480	10.8146
	n	1.2667	0.8667	1.0345	1.0928	1.1445

Thermodynamic parameters including enthalpy (***ΔH***°, kJ mol^{-1}), entropy (***ΔS***°, J·K^{-1}mol^{-1}) and Gibbs free energy (***ΔG***°, kJ mol^{-1}) of the adsorption process can be calculated by **Eq. (5-7).**

$$K_{eq}^{o} = \frac{K_L}{\gamma_{Adsorbate}} \times C^{o} \quad \textbf{(S6)}$$

$$\ln K_{eq}^{o} = \frac{-\Delta G^{o}}{RT} = \frac{\Delta S^{o}}{R} - \frac{\Delta H^{o}}{RT} \quad \textbf{(S7)}$$

$$\Delta G^{o} = \Delta H^{o} - T\Delta S^{o} \quad \textbf{(S8)}$$

where K_{eq}^{o} is the thermodynamic equilibrium constant; K_L (L mg^{-1}) is the Langmuir

equilibrium constant; $C°$= 1 mol L^{-1}; $\gamma_{Adsorbate}$ is the activity coefficient of the adsorbate;

T (K) is the absolute temperature and R (8.314 $J{\cdot}mol^{-1}K^{-1}$) is the ideal gas constant.

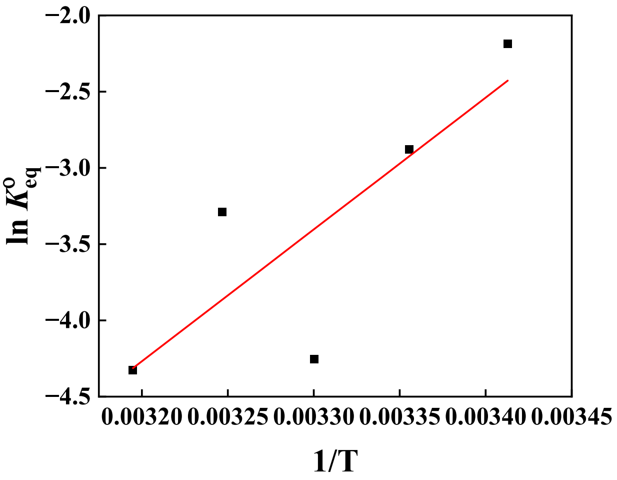

Figure. S3 Experimental data and the fitted curve of InK°_{eq} versus 1/T calculated from van't Hoff plots of adsorption of MB onto GP150

Table S5 Thermodynamic parameters of the adsorption of MB onto GP150

T (K)	Δ$G°$ (kJ mol^{-1})	Δ$H°$ (kJ mol^{-1})	Δ$S°$ ($J{\cdot}K^{-1}mol^{-1}$)
293	-7.729		
298	-7.371		
303	-7.496	-30.832	-80.563
308	-7.620		
313	-7.744		

4. Calibration curves of MB at different pH values

The calibration curves for MB at each pH value were created **(Table S6)**.

Table S6 Calibration curves of MB at different pH values.

pH	calibration curve	R^2
2.24	y= 0.1975x+ 0.0245	0.9989
3.19	y= 0.2007x+ 0.0128	0.9996
4.24	y= 0.2033x+ 0.0099	0.9999
5.23	y= 0.1959x+ 0.0181	0.9996
6.23	y= 0.1991x+ 0.019	1.0000
7.33	y= 0.1977x+ 0.0176	0.9999
8.22	y= 0.1918x+ 0.0182	0.9998
9.24	y= 0.1765x+ 0.0155	0.9996

10.24	y= 0.1791x+ 0.0187	0.9957
